# The association between the type of bystander and survival after an out-of-hospital cardiac arrest: A French nationwide study

**DOI:** 10.1016/j.resplu.2024.100858

**Published:** 2025-01-02

**Authors:** Hizia Benkerrou, Marguerite Lockhart, Matthieu Heidet, Ramy Azzouz, Christian Vilhelm, Hervé Hubert, Morgan Recher, Valentine Baert, GR-RéAC

**Affiliations:** a Univ. Lille, CHU Lille, ULR 2694 - METRICS: Évaluation des Technologies de santé et des Pratiques Médicales, F-59000 Lille, France; b French National Out-of-Hospital Cardiac Arrest Registry, RéAC, F-59000 Lille, France; c Pediatric Intensive Care Unit, CHU Lille, F-59000 Lille, France; d Assistance Publique - Hôpitaux de Paris (AP-HP), SAMU 94 et Urgences, Hôpitaux Universitaires Henri Mondor, F-94000 Créteil, France; e Université Paris-Est Créteil (UPEC), EA-3956 (Control in Intelligent Networks, CIR), F-94000 Créteil, France; f Centre Antipoison et de Toxicovigilance de Lille, CHU Lille, F-59000 Lille, France

**Keywords:** Out-of-hospital cardiac arrest, Cardiopulmonary resuscitation, Basic life support, Bystander interventions

## Abstract

**Background:**

Early bystander interventions are associated with more favorable outcomes after out-of-hospital cardiac arrest (OHCA). The objective of the present study was to determine whether the type of bystander-patient relationship was associated with survival and neurological outcomes after OHCA in France.

**Methods:**

We analyzed data registered in the French National Cardiac Arrest Registry (RéAC) between July 1st, 2011, and April 30th, 2023. The study population comprised bystander-attended cases of OHCA managed by the emergency medical services. Bystanders were categorized as family members, other laypersons, off-duty professional first responders, or off-duty healthcare professionals. The primary outcome was 30-day survival with a favorable neurological outcome (Cerebral Performance Category 1 or 2). The secondary outcomes included the bystander cardiopulmonary resuscitation (CPR) initiation rate, return of spontaneous circulation, and survival upon admission to the hospital. Our statistical analyses were based on bivariate and multivariable logistic regressions analyses.

**Results:**

Among the 89,861 OHCA cases analyzed, family members constituted the largest group of bystanders (69.2%). Compared with non-family-member bystanders, family bystander status was associated with a lower CPR initiation rate, a longer no-flow time, and lower 30-day survival rates. Specifically, cases of OHCA with non-family-member bystanders were 32% more likely to survive with a CPC of 1–2 at day 30 than cases with family member bystanders. Medically trained bystander status (off-duty professional first responders and healthcare professionals) was associated with higher CPR initiation and 30-day survival rates, relative to nontrained laypersons.

**Conclusions:**

Survival after an OHCA appears to be associated with the type of bystander. Although family members were the most common bystanders, they were less likely to initiate CPR and less likely to see the OHCA patient survive. Efforts to increase the post-OHCA survival rate should include targeted interventions (such as public education and training programs) that emphasize the importance of early CPR and automated external defibrillator use by family members.

## Introduction

Out-of-hospital cardiac arrest (OHCA) is a major health issue. The estimated annual incidence of OHCA in Europe is 84.0 per 100,000 person-years, with 30-day survival rates close to 8%.^1^, ^2^ In France, the annual incidence is 61.5 per 100,000 person-years (corresponds to approximately 46,000 OHCAs annually) and the 30-day survival rate is only 5%.^3^ The management of OHCAs is generally based on the “chain of survival” concept, which comprises four steps: (i) early recognition, (ii) early cardiopulmonary resuscitation (CPR), (iii) early defibrillation, and (iv) advanced care.^4^ Bystander CPR is critically associated with favorable outcomes.^5^, ^6^ Given that the no-flow time (i.e. the time interval between collapse and the initiation of CPR) and the time to defibrillation are two of the most important survival factors,^7^, ^8^ bystander intervention is essential before the arrival of an emergency medical services (EMS) unit capable of providing basic life support (BLS) or advanced life support (ALS).^9^, ^10^, ^11^, ^12^, ^13^ However, bystander BLS initiation rates vary markedly from one country to another (e.g. from 13% in Serbia to 46% in France and over 82% in Norway).^2^ Identifying the determinants of this variability might help to increase the frequency of bystander BLS provision. The relationship between the bystander and the patient may be one such determinant. According to the scientific literature, family bystanders rarely provide BLS or provide it with some delay, which reduced the chance of survival,^14^, ^15^, ^16^, ^17^, ^18^, ^19^. However, this factor has not previously been evaluated in France.

Therefore, the primary objective of the present study was to determine whether the type of bystander-victim relationship was associated with survival and neurological outcomes after OHCA in France. The secondary objectives were to assess the association between the bystander-patient relationship and initiation of BLS and to describe time trends in layperson involvement in the response to OHCA.

## Method

### Study setting

In France, the Emergency Medical Services (EMSs) that manage OHCA are two-tiered. The first paramedic response and BLS are usually provided by the fire department. Advanced life support (ALS) is usually then provided by a physician-staffed mobile intensive care unit (MICU), coordinated by a medical dispatch center.^20^ The MICU physician uses a specific, Utstein Style form to collect data on case characteristics, bystander interventions, intervention times, the initiation of BLS and/or ALS, and the patient’s survival status immediately after the cardiac arrest. The data is sent to a secure database managed by the French National Cardiac Arrest Registry (*Registre Électronique des Arrêts Cardiaques* (RéAC); https://www.registreac.org).^21^, ^22^ A follow-up report is filed 30 days after the OHCA or upon hospital discharge.

### Study data

We included patients from the RéAC database for the period of July 1st, 2011, and April 30th, 2023. Included variables regarding the context of the OHCA were, the year of OHCA, the location (at home or other public or private places) of OHCA, the presence of bystander at the time of collapse status (yes/no), the type of bystander (family member, off-duty professional first responder, off-duty healthcare professional, or other bystander). We also assessed the patient’s baseline clinical characteristics, sex, age, medical history, bystander CPR status, first documented rhythm, no-flow and low-flow (time between first resuscitation gesture and ROSC or death) times, the initiation of ALS (including intubation and epinephrine injection), return of spontaneous circulation (ROSC), vital status upon admission to hospital, and 30-day follow-up data.All the definitions of collected data were based on the international Utstein Style template, Data collected according to previous versions of the template have been transformed to follow the new definitions.^22^

### Inclusion and exclusion criteria

We included all OHCAs with one or more bystanders but excluded cases in which the patient had clear signs of death (rigor mortis), patients with unknown cardiac rhythm, cases in which the patient had a normal cardiac rhythm at MICU’s arrival, cases in which the rhythm was not recorded, and OHCAs lacking an obvious medical cause. A medical cause was defined (in line with the Utstein Style guidelines) as a cardiac cause, respiratory disease, neurological disease, anaphylaxis, asthma, and gastrointestinal bleeding. We also excluded cases with a no-flow time greater than 60 min upon the MICU’s arrival and cases with missing data for BLS and 30-day survival.

### Main exposure

The main exposure was the bystander, defined as the first person who was not actively part of an organized emergency response system and who found or witnessed a patient having suffered from an OHCA.^21^ The bystander was classified into four categories: (i) a family member, (ii) an off-duty professional first responder, (iii) an off-duty healthcare professional, or (iv) another layperson (a passerby, a work colleague, etc.). Professional first responders and healthcare professionals were only considered if they were off-duty at the time of the OHCA.

### Outcomes

The primary outcome was 30-day post-OHCA survival with a good neurological outcome (defined as a Cerebral Performance Category (CPC) of 1 or 2).^23^ The secondary outcomes were the bystander CPR initiation rate, ROSC, survival upon admission to hospital, and the overall hospital discharge and 30-day survival rates.

### Statistical analysis

Qualitative variables were described as the frequency (percentages). For quantitative variables, the normality of the data distribution was assessed using the Kolmogorov Smirnov test. Non-normally distributed variables were described as the median andinterquartile range [IQR)]. We compared bystander categories in three sets of analysis, as follows: (i) family members vs. all other bystanders (i.e. non-family-members), (ii) laypersons (family members and others) vs. medically trained persons (off-duty professional first responders and off-duty healthcare professionals), and (iii) family members vs. other laypersons vs. off-duty professional first responders vs. off-duty healthcare professionals.

We also compared the groups with regard to the OHCA context, intervention, and outcome. Univariate analyses were performed using Pearson’s chi-squared test (for qualitative variables) and the Mann-Whitney *U* test or the Kruskal-Wallis test, as appropriate, for quantitative variables. Next, we performed bivariate and multivariate logistic regression analyses of the relationship between each study outcome and the bystander type. For the first two analyses described in the previous paragraph, the reference was “family member”. For the third analysis, “layperson” was used as reference. For the multivariate analyses, we included 12 clinically or methodologically (identified with bivariate analysis) relevant potential confounders: the year of occurrence, sex (male/female/other), age (in years), personal medical history (six variables: heart disease/respiratory disease/diabetes/end-of-life scenario/other/none), no-flow time (in minutes), location (home/public place/healthcare facility), treatment by a MICU (yes/no), and cardiac rhythm on the MICU’s arrival (asystole/pulseless electrical activity/VF-pulseless VT/spontaneous activity). The odds ratio (OR) [95% confidence interval (CI)] was calculated for each variable.

Lastly, we reported time trends in the bystander CPR initiation rate and 30-day (D30) survival according to the bystander type (family member or other) and calculated the range (defined as the difference between the lowest and highest values recorded). We used the Cochran-Armitage test to assess the statistical significance of time trends in the BLS initiation rate.

The threshold for statistical significance in tests was set to p < 0.05. All statistical analyses were performed with R software (version 4.0.3).^24^

### Ethics

In line with the French legislation on retrospective studies of anonymous medical registry data, the study protocol was approved by a hospital committee with competency for research not requiring authorization by an institutional review board. Likewise, patient consent was not required. The study database was registered with the French National Data Protection Commission (*Commission nationale de l'informatique et des libertés* (Paris, France); reference: 910946, dated April 6th, 2012), and the French Advisory Committee on Information Processing in Health Research (*Comité Consultatif sur le Traitement de l’Information en matière de Recherche dans le domaine de la Santé* (Paris, France); reference: 10.326Ter, dated October 14th, 2010).

## Results

**Fig. 1:**
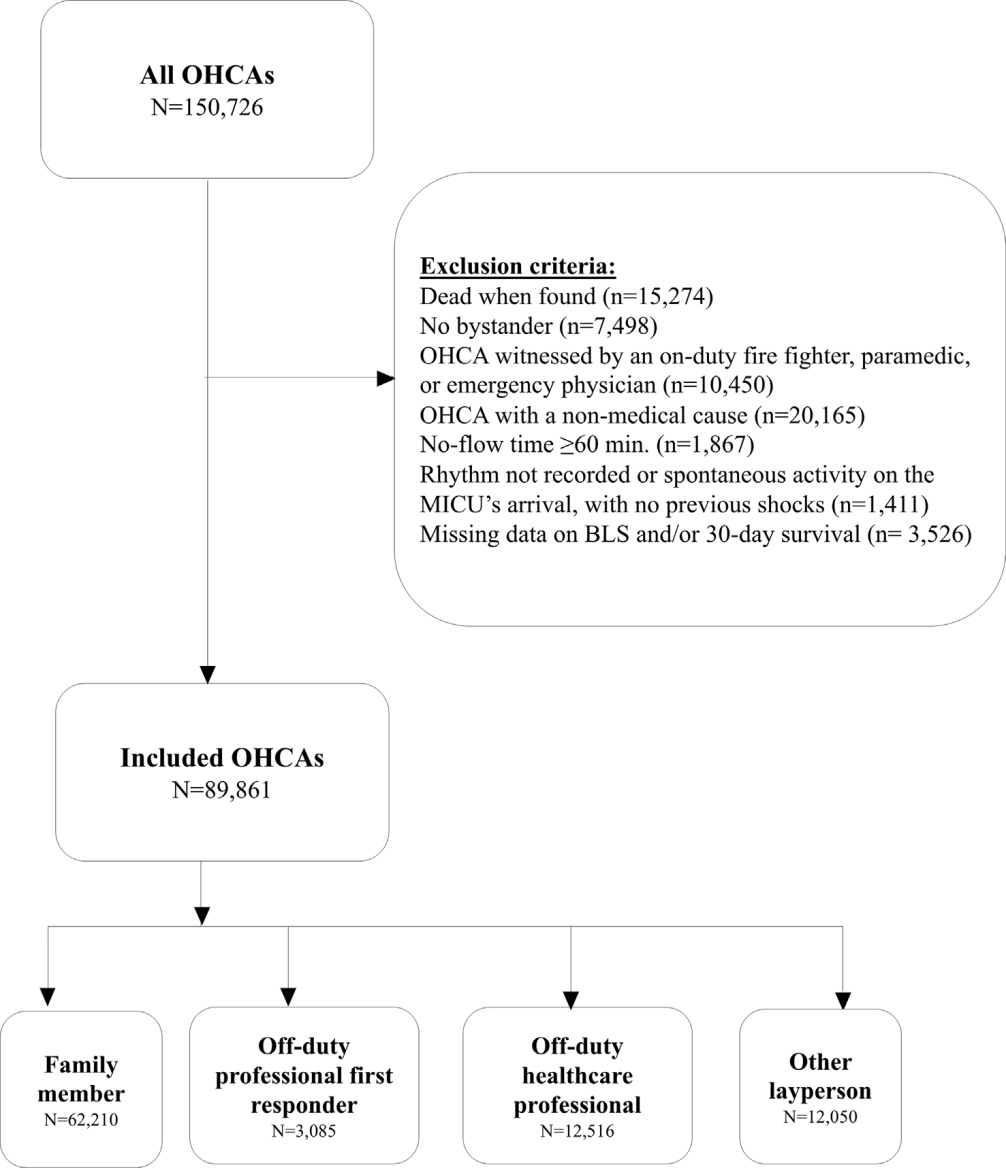
Study flowchart.

From 2011 to 2023, 150,726 patients were included in the RéAC database. Of these, 60,865 met at least one exclusion criterion, and so 89,861 patients were included in the present analysis (Table 1): 62,210 (69.2%) in the family member group, 3,085 (3.4%) in the off-duty professional first responder group, 12,516 (13.9%) in the off-duty healthcare professional bystander group, and 12,050 (13.4%) in the other layperson group (Fig. 1).

**Table 1:** Characteristics of the study population.

	Study population (n = 89,861)
Cases per year	
2011	493 (0.5)
2012	4462 (5.0)
2013	8778 (9.8)
2014	8677 (9.7)
2015	9264 (10.3)
2016	8779 (9.8)
2017	8848 (9.8)
2018	8781 (9.8)
2019	8423 (9.4)
2020	8141 (9.1)
2021	7387 (8.2)
2022	6431 (7.2)
2023	1397 (1.6)
Sex (male)	59,048 (65.7)
Age (years)	71 [59; 82]
Medical history	
Heart disease	40,975 (45.6)
Respiratory disease	12,983 (14.4)
Diabetes	12,835 (14.3)
End-of-life scenario	3113 (3.5)
Other	30,100 (33.5)
None	7885 (8.8)
OHCA location (home)	64,965 (77.5)
Witnessed OHCA	62,421 (69.5)
No-flow time (min)	10 [4; 18]
Bystander CPR	47,042 (52.3)
Bystander type	
Family	62,210 (69.2)
Other layperson	12,050 (13.4)
Off-duty professional first responder	3085 (3.4)
Off-duty healthcare professional	12,516 (13.9)
CPR provided by the bystander	
Ventilation only	194 (0.4)
Chest compression + ventilation	10,954 (24.1)
Chest compression only	34,319 (75.5)
AED application by the bystander	7467 (11.2)
Bystander AED shock when applied	2405 (35.3)
First MICU-recorded cardiac rhythm	
Asystole	72,081 (80.9)
PEA	6115 (6.9)
VF/pulseless VT	7708 (8.6)
ROSC	3221 (3.6)
MICU-treated	64,931 (72.3)
Tracheal intubation	59,794 (66.5)
Epinephrine administration	58,335 (65.0)
Low-flow time (min)	29 [16; 42]
ROSC	19,005 (21.1)
Alive on arrival at hospital	15,860 (17.6)
Alive at 30 days	4348 (4.8)
CPC 1–2 at 30 days	3337 (3.7)

Results are presented as frequency (percentage) or mediane [IQR].

* The year 2011 was from July to December and the year 2023 was from January to April.

AED: automated external defibrillator; BLS: basic life support; CPR: bystander cardiopulmonary resuscitation; CPC: Cerebral Performance Category; MICU: mobile intensive care unit; OHCA: out-of-hospital cardiac arrest; PEA: pulseless electrical activity; ROSC: return of spontaneous circulation; VF/pulseless VT: ventricular fibrillation/pulseless ventricular tachycardia.

Baseline characteristics of the population are described in the Table 1. The majority of OHCA patients were male (59,048 [65.7%]), and the median [IQR] age was 71 [59; 82]. In the majority of cases (62,421 [69.5%]), a bystander was present at the time of collapse. Bystanders initiated CPR in 47,042 (52.3%) cases, and an automated external defibrillator (AED) was applied in 7,467 (11.2%) cases. The median [IQR] no-flow time was 10 [4; 18] minutes, and the first rhythm recorded on the MICU’s arrival was asystole in 72,081 (80.9%) cases. ALS was provided in 64,931 (72.3%) cases, and the median [IQR] low-flow time was 29 [16; 42] minutes. ROSC was observed in 19,005 (21.1%) cases, 15,860 (17.6%) cases were alive on arrival at hospital, and 4,348 (4.8%) were alive 30 days after OHCA or on discharge from hospital (Table 1).

### Patient characteristics, according to the type of bystander: Univariate analysis

#### Family member bystanders vs. all other bystanders

In this first comparison, we compared 62,210 family member bystanders with 27,651 non-family-member bystanders (Supplementary Table 1). For a family member bystander, the OHCA patient was more likely to be male (41,214 [66.2%] vs. 17,834 [64.5%] for a non-family-member bystander, p < 0.001), the OHCA was more likely to occur at home (54,649 [94.2%] vs. 10,316 [40.0%], respectively, p < 0.001) and less likely to be witnessed (42,268 [67.9%] vs. 20,153 [72.9%], respectively, p < 0.001). Lastly, a family member bystander was less likely to perform CPR (27,320 [43.9%] vs. 19,722 [71.3%] for a non-family-member bystander, p < 0.001), and OHCA patients with a family member bystander had longer no-flow times (11 [5; 18] minutes vs. 9 [1; 17] minutes for a non-family-member bystander, p < 0.001). In the group of patients with a family member as a witness, the use of an AED was less frequent than in the non-family member group (5.2% vs. 24.3%, p < 0.001) and these patients were less likely to achieve a good 30-day neurologic outcome (1,500 [2.4%] vs. 1,837 [6.7%], respectively, p < 0.001).

#### Laypersons vs. medically trained bystanders

We next compared 74,260 layperson bystanders with 15,601 medically trained bystanders (Supplementary Table 2). For a layperson bystander, the OHCA patient was more likely to be male (50,286 [67.7%] vs. 8,762 [56.2%] for a medically trained bystander, p < 0.001), and the OHCA was less likely to have been witnessed (50,023 [67.4%] vs. 12,398 [79.5%], respectively, p < 0.001). A layperson bystander was less likely to perform CPR (34,130 [46.0%] vs. 12,912 [82.8%] for a medically trained bystander, p < 0.001). OHCA patients with a layperson bystander had a lower 30-day CPC1/2 survival rate than patients with a (medically trained bystander (respectively 2,451 [3.3%] vs. 886 [5.7%], p < 0.001).

#### Family members vs. other laypersons vs. off-duty professional first responders vs. off-duty healthcare professionals

We next compared family member bystanders (n = 62,210) with other lay bystanders (n = 12,050), off-duty professional first responders (n = 3,085), and off-duty healthcare professionals (n = 12,516). (Supplementary Table 3). The OHCA was less likely to have been witnessed when the bystander was non-family layperson (7,755 [64.4%], vs. 42,268 [67.9%] for family members, 2,657 [86.1%] for off-duty professional first responders, and 9,741 [77.8%] for off-duty healthcare professionals, p < 0.001). Family member bystanders were the least willing to perform CPR (27,320 [43.9%], vs. 6,810 [56.5%] for other laypersons, 2,792 [90.5%] for off-duty professional first responders, and 10,120 [80.9%] for off-duty healthcare professionals, p < 0.001). The 30-day CPC1/2 survival rate was lowest for family member bystanders in this group (1,500 [2.4%], vs. 951 [8.0%] for other laypersons, 311 [10.2%] for off-duty professional first responders, and 575 [4.6%] for off-duty healthcare professionals, p < 0.001).

### Bystander type and survival outcomes

#### Family member bystanders vs. all other bystanders

In the first regression model, we compared family member bystanders with all other bystanders (Table 2). In our adjusted analyses, the presence of a non-family-member bystander had higher odds ratio ofCPR initiation (OR = 2.79 [2.68–2.91]), more likely ROSC (OR = 1.12 [1.06–1.18]), and higher odds ratio of survival at admission to hospital (OR = 1.19 [1.12–1.25]).

**Table 2:** Bivariate and multivariable logistic regression analyses of outcomes by type of bystander (family vs. non-family).

Outcome	Study group	Bivariate model OR [95% CI]	Multivariate model* OR [95% CI]
CPC 1–2 at 30 days			
	Family	*ref.*	*ref.*
	Non-family	2.90 [2.70; 3.11]	1.32 [1.18; 1.48]
Bystander CPR			
	Family	*ref.*	*ref.*
	Non-family	3.18 [3.08; 3.28]	2.79 [2.68; 2.91]
ROSC			
	Family	*ref.*	*ref.*
	Non-family	1.51 [1.46; 1.56]	1.12 [1.06; 1.18]
Alive on arrival at hospital			
	Family	*ref.*	*ref.*
	Non-family	1.72 [1.66; 1.78]	1.19 [1.12; 1.25]
Alive at 30-day			
	Family	*ref.*	*ref.*
	Non-family	2.76 [2.59; 2.93]	1.27 [1.15; 1.41]

* adjusted for year, sex, age, medical history, location, no-flow time, MICU-treated and first MICU-recorded cardiac rhythm.

CI: confidence interval; CPC: Cerebral Performance Category; CPR: cardiopulmonary resuscitation; OR: odds-ratio; ROSC: return of spontaneous circulation.

#### Laypersons vs. medically trained bystanders

In the second regression model, we compared layperson bystanders with medically trained bystanders (Table 3). Our adjusted analyses showed that the presence of a medically trained bystander was associated with higher survival rates. The presence of a medically trained bystander was associated with higher odds ratio of CPR initiation and ROSC (OR = 7.38 [6.96–7.84]), ROSC (OR = 1.22 [1.15–1.29]) and higher odds ratio of survival upon admission to hospital (OR = 1.28 [1.20–1.36]). Patients with a medically trained bystander had better odds ratio of CPC1/2 at D30 than those who did not (OR = 1.69 [1.50–1.89]).

**Table 3:** Bivariate and multivariable logistic regression analyses of outcomes by type of bystander (layperson vs. medically trained).

Outcome	Study group	Bivariate model OR [95%CI]	Multivariate model* OR [95%CI]
CPC 1–2 at 30 days			
	Layperson	*ref.*	*ref.*
	Medically trained	1.77 [1.63; 1.91]	1.69 [1.50; 1.89]
Bystander CPR			
	Layperson	*ref.*	*ref.*
	Medically trained	5.65 [5.40; 5.90]	7.38 [6.96; 7.84]
ROSC			
	Layperson	*ref.*	*ref.*
	Medically trained	1.22 [1.18; 1.28]	1.22 [1.15; 1.29]
Alive on arrival at hospital			
	Layperson	*ref.*	*ref.*
	Medically trained	1.22 [1.17; 1.27]	1.28 [1.20; 1.36]
Alive at 30-day			
	Layperson	*ref.*	*ref.*
	Medically trained	1.64 [1.53; 1.76]	1.54 [1.39; 1.72]

* adjusted for year, sex, age, medical history, location, no-flow time, MICU-treated and first MICU-recorded cardiac rhythm.

CI: confidence interval; CPC: Cerebral Performance Category; CPR: cardiopulmonary resuscitation; OR: odds-ratio; ROSC: return of spontaneous circulation.

#### Family members vs. other laypersons vs. off-duty professional first responders vs. off-duty healthcare professionals

In the third and last regression model, we compared four bystander groups: family members vs. other laypersons vs. off-duty professional first responders vs. off-duty healthcare professionals (Table 4). Our adjusted analyses showed that when compared with family members, the presence of off-duty professional first responders or off-duty healthcare professionals was associated with greater survival rates. Patients with an off-duty professional first responder or off-duty healthcare professional bystander had better odds ratio of CPC1/2 at D30 than those with other bystander types (OR = 1.76 [1.45–2.13] and 1.67 [1.44–1.94], respectively). The presence of an off-duty professional first responder bystander or an off-duty healthcare professional bystander was associated with higher odds ratio od CPR initiation (OR = 14.75 [12.87–16.96] and 6.38 [5.98–6.81], respectively) and higher rate of survival upon admission to hospital (OR = 1.45 [1.30–1.61] and 1.25 [1.15–1.35], respectively).

**Table 4:** Bivariate and multivariable logistic regression analyses of the outcome by type of bystander (family vs. off-duty professional first responder vs. off-duty healthcare professional vs. other layperson).

Outcome	Study group	Bivariate model OR [95%CI]	Multivariate model* OR [95%CI]
CPC 1–2 at 30 days			
	Family	*ref.*	*ref.*
	Off-duty professional first responder	4.57 [4.01; 5.19]	1.76 [1.45; 2.13]
	Off-duty Health. Pro	1.95 [1.77; 2.15]	1.67 [1.44; 1.94]
	Other layperson	3.50 [3.22; 3.80]	1.02 [0.89; 1.17]
Bystander CPR			
	Family	*ref.*	*ref.*
	Off-duty professional first responder	12.17 [10.80; 13.77]	14.75 [12.87; 16.96]
	Off-duty Health. Pro	5.36 [5.15; 5.66]	6.38 [5.98; 6.81]
	Other layperson	1.66 [1.60; 1.73]	1.10 [1.04; 1.16]
ROSC			
	Family	*ref.*	*ref.*
	Off-duty professional first responder	1.98 [1.83; 2.14]	1.30 [1.17; 1.44]
	Off-duty Health. Pro	1.22 [1.16; 1.28]	1.19 [1.11; 1.28]
	Other layperson	1.73 [1.65; 1.81]	1.02 [0.96; 1.09]
D0 Survival			
	Family	*ref.*	*ref.*
	Off-duty professional first responder	2.32 [2.14; 2.51]	1.45 [1.30; 1.61]
	Off-duty Health. Pro	1.23 [1.17; 1.29]	1.25 [1.15; 1.35]
	Other layperson	2.14 [2.04; 2.24]	1.08 [1.01; 1.15]
D30 survival			
	Family	*ref.*	*ref.*
	Off-duty professional first responder	4.03 [3.58; 4.54]	1.59 [1.33; 1.89]
	Off-duty Health. Pro	1.84 [1.68; 2.00]	1.56 [1.36; 1.79]
	Other layperson	3.44 [3.20; 3.70]	1.04 [0.92; 1.17]

* adjusted for year, sex, age, medical history, location, no-flow time, MICU-treated and first MICU-recorded cardiac rhythm.

CI: confidence interval; CPC: Cerebral Performance Category; CPR: cardiopulmonary resuscitation; OR: odds-ratio; ROSC: return of spontaneous circulation.

When compared with family members, the “other lay bystander” class was not associated with higher odds ratio of D30 CPC1/2 survival (OR = 1.02 [0.89–1.17]) but was associated with higher offs ratioof CPR initiation (OR = 1.10 [1.04–1.16]) and higher odds ratio of admission to hospital (OR = 1.08 [1.01–1.15]).

### Time trends in bystander involvement

**Fig. 2:**
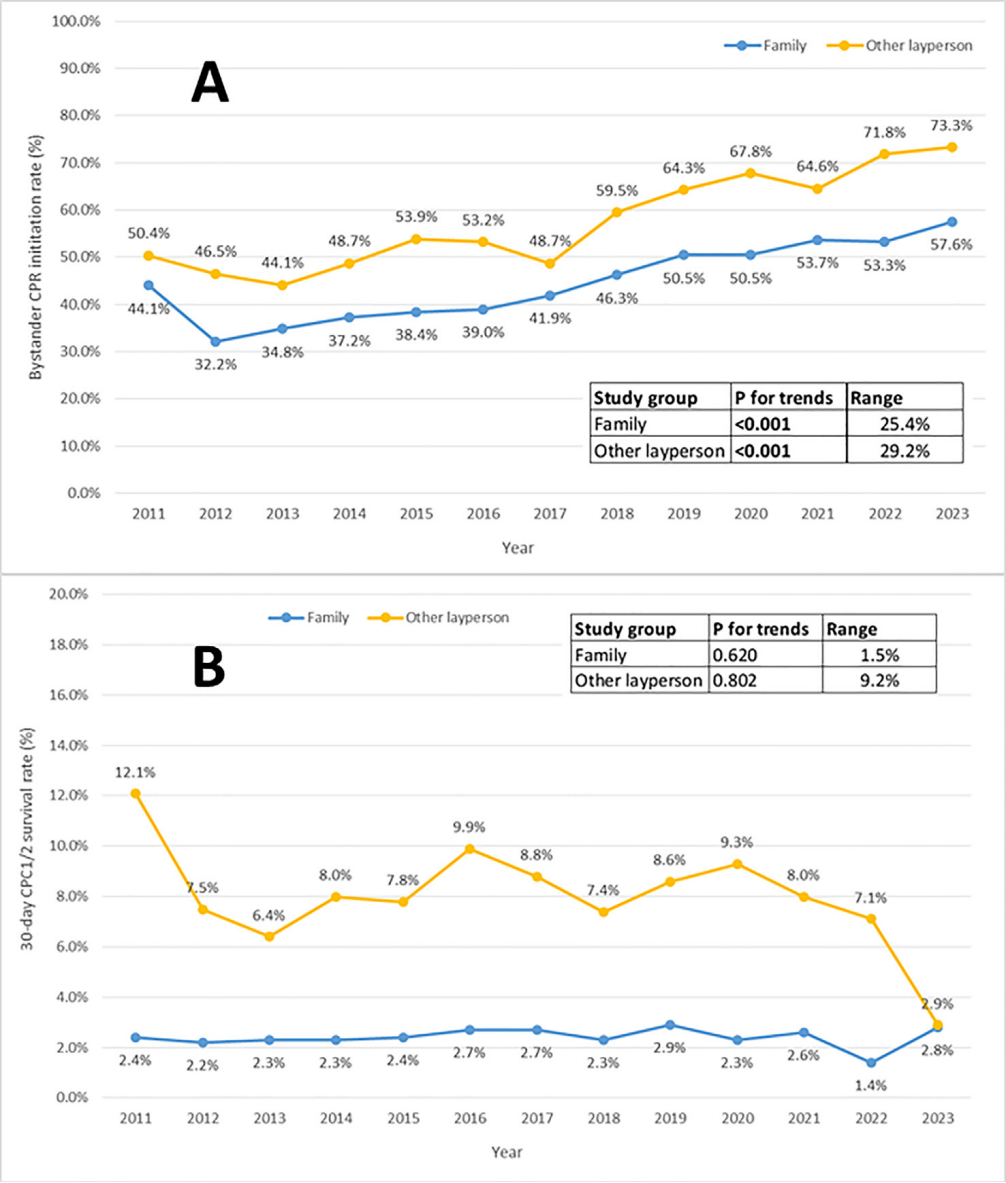
A: Trends in the rate of bystander CPR initiation by type of bystander/ B: Time trends in the 30-day CPC1/2 survival rate, by type of bystander.

We analyzed time trends in CPR initiation and 30-day CPC1/2 survival rates with regard to family members and other layperson (Fig. 2.A and Fig. 2.B, respectively). The family member bystander group was associated with a significant increase over time in the CPR initiation rate; the range was 25.4 percentage points. However, the increase over time in the 30-day CPC1/2 survival rate was not significant (p = 0.620, range = 1.5 percentage points). In the other layperson group, we observed a significant increase over time in the CPR initiation rate; the range was 29.2 percentage points. However, increase over time in the 30-day CPC1/2 survival rate was not significant (p = 0.802, range = 9.2 percentage points).

## Discussion

Based on data drawn from a nationwide, retrospective, observational cohort of OHCAs in France, our present findings indicate that patients who suffered from OHCA in presence of a non-family-member bystander were more likely to survive with a favorable neurological status at 30 days. Moreover, patients of OHCAs in the presence of professional first responder and healthcare professional bystanders were more likely to survive with a favorable neurological status at 30 days, relative to patients with a family-related bystander.

In line with several other studies, we found that family member bystanders are least likely to provide CPR.^6^, ^19^, ^25^, ^26^ There are several possible explanations for this low rate. Psychological factors can significantly influence a person's response to a cardiac arrest: family members witnessing a cardiac arrest by their loved one may experience shock, fear, or disbelief.^27^, ^28^, ^29^, ^30^ These emotional factors might cause the bystander to fail to recognize the severity of the situation and might prevent them from taking effective, immediate action. In contrast, non-family members are less likely to be burdened by these intense emotional factors and so are more likely to act swiftly.

We found that family member bystanders were the least likely to use an AED. Again, there are several possible explanations for this reluctance. The scientific literature shows that when the bystander is a family member, the OHCA is more likely to have occurred at home (i.e. a non-public place where an AED is less likely to be available).^26^, ^29^, ^30^, ^31^, ^32^, ^33^, ^34^ All these factors contribute to lower survival rates with family member bystanders. Indeed, our adjusted analysis revealed a 32% greater chance of 30-day CPC1/2 survival when the bystander was not family-related. Several studies have found similar results,^14^, ^15^, ^17^, ^19^ however, the most frequent bystanders in our study were family members (69.2%). This observation is in line with the scientific literature again and show that family members should be trained to be effective OHCA witness.^19^, ^29^, ^35^

The time-trend analysis showed a significant increase in the frequency of CPR initiation by a family member bystander, with a rise between 2012 and2023. At first sight, these results are quite encouraging. However, we did not observe a statisticalsignificance change over time in the 30-day CPC1/2 survival, with a range of 1.5%. Those two observations raise questions about the time to BLS initiation and the quality of the BLS initiation, since the initiation of BLS is known to be the most important factor in OHCA survival.^5^, ^6^, ^9^, ^11^ Indeed, it is reasonable to assume that the time to BLS initiation and the quality of the BLS initiation are at least partly responsible for this discrepancy. One possible explanation for the increase over the last decade in the frequency of BLS initiation by family members could be that dispatching centers have been widely encouraged and trained to provide telephone guidance on CPR to bystanders at the scene of the OHCA.^36^, ^37^ This telephone guidance might encourage the bystander to perform CPR but, as reported previously, has not yet had an impact on survival rates − probably due to the poor quality of CPR initiation.^38^ Another possible explanation is the long time to BLS initiation. Indeed, it has been reported previously that the no-flow time is one of the most important survival factors for OHCAs.^7^, ^8^ In this line, a study found that the no-flow time during layperson CPR has increased significantly over time.^39^ These results should be the subject of further investigation. Furthermore, prior training and experience in emergency response might be more common among non-family members and might contribute to the observed difference in survival outcomes. Individuals who have received BLS training or have already handled an emergency situation might feel more confident and competent in performing CPR and in calling for professional assistance. In contrast, family members who lack such training might be hesitant or unsure of the appropriate actions to be taken in the critical moments following a cardiac arrest.^28^, ^29^ Our results support the hypothesis whereby training helps to increase the chances of survival: the likelihood of 30-day CPC1/2 survival was 69% greater when the bystander was medically trained, compared with a layperson.^18^, ^40^

Our findings indicate that public health strategies aimed at improving cardiac arrest survival rates must be modified profoundly. It is clear that providing BLS training to the general public (e.g. the “Kids Save Lives” project promoted by the European Resuscitation Council) is of paramount importance.^41^, ^42^, ^43^ The “Kids Save Lives” project seeks to provide schoolchildren with BLS as early as possible in their education. Efforts must focus on educating the general public about the importance of CPR training and early use of an AED.^43^, ^44^, ^45^ Providing children with training is likely to be the only way of achieving sufficient national coverage in BLS competency. By implementing this type of initiative on a national level, we'll be able to have, within a few decades, a national population fully trained in life-saving gestures, and thus maximize our chances of having effective witnesses.

### Strengths and limitations

This study had several limitations, including its internal validity and comprehensive dataset. During a 12-year period, more than 150,000 all-cause OHCAs have been recorded in the RéAC database. A low proportion of missing data was guaranteed by the various quality controls implemented at the local dispatch centers and at the national level. To the best of our knowledge, the present study is the first in France to have examined the relationship between bystander status and OHCA cases.

The study also had some limitations. The study data were collected retrospectively, and certain confounding variables might not have been accounted for fully. Secondly, the study focused solely on the relationship ofthe first bystander and the patient, without considering the actions potentially taken by other bystanders. Future research should explore the influence of specific interventions performed by different types of bystander, in order to gain a more comprehensive understanding of the relationships with survival outcomes. Lastly, our study included pre-hospital care but not consider post-resuscitation care, it can affect the survival rates.

## Conclusion

In a nationwide study conducted in France, the CPR rate and clinical OHCA outcomes varied with the type of bystander. The presence of a family member as the first bystander was associated with a lower 30-day survival rate. Although family members tend to initiate resuscitation more frequently over the years, this is not associated with increased survival. Our findings emphasize the need for targeted interventions, including education and training programs for the general public.

## CRediT authorship contribution statement

**Hizia Benkerrou:** Writing – original draft, Methodology, Formal analysis, Conceptualization. **Marguerite Lockhart:** Writing – review & editing, Validation, Methodology, Investigation. **Matthieu Heidet:** Writing – review & editing, Validation, Investigation. **Ramy Azzouz:** Writing – review & editing, Investigation, Formal analysis. **Christian Vilhelm:** Writing – review & editing, Validation, Software, Investigation, Data curation. **Hervé Hubert:** Writing – review & editing, Supervision, Methodology, Investigation, Data curation, Conceptualization. **Morgan Recher:** Writing – review & editing, Supervision, Methodology, Investigation. **Valentine Baert:** Writing – review & editing, Validation, Supervision, Methodology, Formal analysis, Data curation, Conceptualization.

## Declaration of competing interest

The authors declare that they have no known competing financial interests or personal relationships that could have appeared to influence the work reported in this paper.
