# Supplementary material for: The association between the type of bystander and survival after an out-of-hospital cardiac arrest: A French nationwide study
Source: Resusc Plus. 2025 Jan 2;21:100858. doi: 10.1016/j.resplu.2024.100858 (PMC11780125; doi:10.1016/j.resplu.2024.100858)
Supplement: Supplementary Data 1 [file mmc1.docx]

Supplementary Table 1: Characteristics of the population by type of bystander (family vs. non-family).

| **Characteristics** | | Family | Non-family | **p-value** |
| --- | --- | --- | --- | --- |
|  |  | (n = 62,210) | (n = 27,651) |  |
| Cases per year* | |  |  | **<0.001** |
|  | 2011 | 295 (0.5) | 198 (0.7) |  |
|  | 2012 | 2940 (4.7) | 1522 (5.5) |  |
|  | 2013 | 5732 (9.2) | 3046 (11.0) |  |
|  | 2014 | 5663 (9.1) | 3014 (10.9) |  |
|  | 2015 | 6412 (10.3) | 2852 (10.3) |  |
|  | 2016 | 6112 (9.8) | 2667 (9.6) |  |
|  | 2017 | 6188 (9.9) | 2660 (9.6) |  |
|  | 2018 | 6061 (9.7) | 2720 (9.8) |  |
|  | 2019 | 5882 (9.5) | 2541 (9.2) |  |
|  | 2020 | 6005 (9.7) | 2136 (7.7) |  |
|  | 2021 | 5339 (8.6) | 2048 (7.4) |  |
|  | 2022 | 4590 (7.4) | 1841 (6.7) |  |
|  | 2023 | 991 (1.6) | 406 (1.5) |  |
| Sex (male) | | 41214 (66.2) | 17834 (64.5) | **<0.001** |
| Age (years) | | 71 [60; 81] | 71 [58; 84] | **<0.001** |
| Medical history | |  |  |  |
|  | Heart disease | 29319 (47.1) | 11656 (42.2) | **<0.001** |
|  | Respiratory disease | 9635 (15.5) | 3348 (12.1) | **<0.001** |
|  | Diabetes | 9364 (15.1) | 3471 (12.6) | **<0.001** |
|  | End-of-life scenario | 2358 (3.8) | 755 (2.7) | **<0.001** |
|  | Other | 20846 (33.5) | 9254 (33.5) | 0.908 |
|  | None | 4763 (7.7) | 3122 (11.3) | **<0.001** |
| OHCA location (home) | | 54649 (94.2) | 10316 (40.0) | **<0.001** |
| Witnessed OHCA | | 42268 (67.9) | 20153 (72.9) | **<0.001** |
| No-flow time (min) | | 11 [5; 18] | 9 [1; 17] | **<0.001** |
| Bystander CPR | | 27320 (43.9) | 19722 (71.3) | **<0.001** |
| Bystander CPR type | |  |  | **<0.001** |
|  | Ventilation only | 136 (0.5) | 58 (0.3) |  |
|  | Chest compression + ventilation | 3688 (14.0) | 7266 (37.8) |  |
|  | Chest compression only | 22428 (85.4) | 11891 (61.9) |  |
| Bystander AED application | | 2374 (5.2) | 5093 (24.3) | **<0.001** |
| Bystander AED shock when applied | | 694 (31.4) | 1711 (37.2) | **<0.001** |
| First MICU-recorded cardiac rhythm | |  |  | **<0.001** |
|  | Asystole | 51612 (83.5) | 20469 (74.9) |  |
|  | PEA | 3837 (6.2) | 2278 (8.3) |  |
|  | VF/pulseless VT | 4845 (7.8) | 2863 (10.5) |  |
|  | ROSC | 1486 (2.4) | 1735 (6.3) |  |
| MICU-treated | | 44909 (72.2) | 20022 (72.4) | 0.501 |
| Tracheal intubation | | 41248 (66.3) | 18546 (67.1) | **0.025** |
| Epinephrine administration | | 40820 (65.7) | 17515 (63.5) | **<0.001** |
| Low-flow time (min) | | 30 [17; 42] | 28 [15; 42] | **<0.001** |
| ROSC | | 11785 (18.9) | 7220 (26.1) | **<0.001** |
| Alive on arrival at hospital | | 9393 (15.1) | 6467 (23.4) | **<0.001** |
| Alive at 30-day | | 2013 (3.2) | 2335 (8.4) | **<0.001** |
| CPC 1-2 at 30-day | | 1500 (2.4) | 1837 (6.7) | **<0.001** |
| * Year 2011 is from July to December and year 2023 is from January to April. | | | |  |
| AED: automated external defibrillator; BLS: basic life support; CPR: bystander cardiopulmonary resuscitation; CPC: Cerebral Performance Category; MICU: mobile intensive care unit; OHCA: out-of-hospital cardiac arrest; PEA: pulseless electrical activity; ROSC: return of spontaneous circulation; VF/pulseless VT: ventricular fibrillation/pulseless ventricular tachycardia. | | | | |

Supplementary Table 2: Characteristics of the population by type of bystander (layperson vs. medically trained).

| **Characteristics** | | Layperson | Medically trained | **p-value** |
| --- | --- | --- | --- | --- |
|  |  | (n = 74,260) | (n = 15,601) |  |
| Cases per year* | |  |  | **<0.001** |
|  | 2011 | 412 (0.6) | 81 (0.5) |  |
|  | 2012 | 3780 (5.1) | 682 (4.4) |  |
|  | 2013 | 7160 (9.6) | 1618 (10.4) |  |
|  | 2014 | 7131 (9.6) | 1546 (9.9) |  |
|  | 2015 | 7605 (10.2) | 1659 (10.6) |  |
|  | 2016 | 7188 (9.7) | 1591 (10.2) |  |
|  | 2017 | 7218 (9.7) | 1630 (10.4) |  |
|  | 2018 | 7153 (9.6) | 1628 (10.4) |  |
|  | 2019 | 6894 (9.3) | 1529 (9.8) |  |
|  | 2020 | 6923 (9.3) | 1218 (7.8) |  |
|  | 2021 | 6259 (8.4) | 1128 (7.2) |  |
|  | 2022 | 5370 (7.2) | 1061 (6.8) |  |
|  | 2023 | 1167 (1.6) | 230 (1.5) |  |
| Sex (male) | | 50286 (67.7) | 8762 (56.2) | **<0.001** |
| Age (years) | | 70 [58; 81] | 77 [63; 86] | **<0.001** |
| Medical history | |  |  |  |
|  | Heart disease | 33464 (45.1) | 7511 (48.1) | **<0.001** |
|  | Respiratory disease | 10697 (14.4) | 2286 (14.7) | 0.430 |
|  | Diabetes | 10468 (14.1) | 2367 (15.2) | **<0.001** |
|  | End-of-life scenario | 2497 (3.4) | 616 (3.9) | **<0.001** |
|  | Other | 24184 (32.6) | 5916 (37.9) | **<0.001** |
|  | None | 6962 (9.4) | 923 (5.9) | **<0.001** |
| OHCA location (home) | | 58692 (84.6) | 6273 (43.5) | **<0.001** |
| Witnessed OHCA | | 50023 (67.4) | 12398 (79.5) | **<0.001** |
| No-flow time (min) | | 10 [5; 17] | 10 [1; 18] | **<0.001** |
| Bystander CPR | | 34130 (46.0) | 12912 (82.8) | **<0.001** |
| Bystander CPR type | |  |  | **<0.001** |
|  | Ventilation only | 146 (0.4) | 48 (0.4) |  |
|  | Chest compression + ventilation | 4664 (14.2) | 6290 (49.4) |  |
|  | Chest compression only | 27925 (85.3) | 6394 (50.2) |  |
| Bystander AED application | | 3727 (6.8) | 3740 (32.7) | **<0.001** |
| Bystander AED shock when applied | | 1375 (39.7) | 1030 (30.8) | **<0.001** |
| First MICU-recorded cardiac rhythm | |  |  | **<0.001** |
|  | Asystole | 60204 (81.7) | 11877 (77.0) |  |
|  | PEA | 4731 (6.4) | 1384 (9.0) |  |
|  | VF/pulseless VT | 6516 (8.8) | 1192 (7.7) |  |
|  | ROSC | 2241 (3.0) | 980 (6.4) |  |
| MICU-treated | | 54729 (73.7) | 10202 (65.4) | **<0.001** |
| Tracheal intubation | | 50553 (68.1) | 9241 (59.2) | **<0.001** |
| Epinephrine administration | | 49572 (66.8) | 8763 (56.4) | **<0.001** |
| Low-flow time (min) | | 30 [17; 42] | 26 [14; 40] | **<0.001** |
| ROSC | | 15253 (20.5) | 3752 (24.0) | **<0.001** |
| Alive on arrival at hospital | | 12719 (17.1) | 3141 (20.1) | **<0.001** |
| Alive at 30-day | | 3257 (4.4) | 1091 (7.0) | **<0.001** |
| CPC 1-2 at 30-day | | 2451 (3.3) | 886 (5.7) | **<0.001** |
|  |  |  |  |  |
| * Year 2011 is from July to December and year 2023 is from January to April. | | | |  |
| AED: automated external defibrillator; BLS: basic life support; CPR: bystander cardiopulmonary resuscitation; CPC: Cerebral Performance Category; MICU: mobile intensive care unit; OHCA: out-of-hospital cardiac arrest; PEA: pulseless electrical activity; ROSC: return of spontaneous circulation; VF/pulseless VT: ventricular fibrillation/pulseless ventricular tachycardia. | | | | |

Supplementary Table 3: Characteristics of the population by type of bystander (family vs. other layperson vs. off-duty professional first responder vs. off-duty healthcare professional).

| **Characteristics** | | Family | Other layperson | Off-duty professional first responder | Off-duty healthcare professional | **p-value** |
| --- | --- | --- | --- | --- | --- | --- |
|  |  | (n = 62,210) | (n = 12,050) | (n = 3,085) | (n = 12,516) |  |
| Cases per year* | |  |  |  |  | **<0.001** |
|  | 2011 | 295 (0.5) | 117 (1.0) | 9 (0.3) | 72 (0.6) |  |
|  | 2012 | 2940 (4.7) | 840 (7.0) | 113 (3.7) | 569 (4.5) |  |
|  | 2013 | 5732 (9.2) | 1428 (11.9) | 237 (7.7) | 1381 (11.0) |  |
|  | 2014 | 5663 (9.1) | 1468 (12.2) | 244 (7.9) | 1302 (10.4) |  |
|  | 2015 | 6412 (10.3) | 1193 (9.9) | 315 (10.2) | 1344 (10.7) |  |
|  | 2016 | 6112 (9.8) | 1076 (8.9) | 303 (9.8) | 1288 (10.3) |  |
|  | 2017 | 6188 (9.9) | 1030 (8.5) | 353 (11.4) | 1277 (10.2) |  |
|  | 2018 | 6061 (9.7) | 1092 (9.1) | 349 (11.3) | 1279 (10.2) |  |
|  | 2019 | 5882 (9.5) | 1012 (8.4) | 333 (10.8) | 1196 (9.6) |  |
|  | 2020 | 6005 (9.7) | 918 (7.6) | 262 (8.5) | 956 (7.6) |  |
|  | 2021 | 5339 (8.6) | 920 (7.6) | 264 (8.6) | 864 (6.9) |  |
|  | 2022 | 4590 (7.4) | 780 (6.5) | 253 (8.2) | 808 (6.5) |  |
|  | 2023 | 991 (1.6) | 176 (1.5) | 50 (1.6) | 180 (1.4) |  |
| Sex (male) | | 41214 (66.2) | 9072 (75.3) | 2084 (67.6) | 6678 (53.4) | **<0.001** |
| Age (years) | | 71 [60; 81] | 65 [53; 78] | 69 [56; 81] | 79 [66; 87] | **<0.001** |
| Medical history | |  |  |  |  |  |
|  | Heart disease | 29319 (47.1) | 4145 (34.4) | 1167 (37.8) | 6344 (50.7) | **<0.001** |
|  | Respiratory disease | 9635 (15.5) | 1062 (8.8) | 330 (10.7) | 1956 (15.6) | **<0.001** |
|  | Diabetes | 9364 (15.1) | 1104 (9.2) | 371 (12.0) | 1996 (15.9) | **<0.001** |
|  | End-of-life scenario | 2358 (3.8) | 139 (1.2) | 69 (2.2) | 547 (4.4) | **<0.001** |
|  | Other | 20846 (33.5) | 3338 (27.7) | 866 (28.1) | 5050 (40.3) | **<0.001** |
|  | None | 4763 (7.7) | 2199 (18.2) | 306 (9.9) | 617 (4.9) | **<0.001** |
| OHCA location (home) | | 54649 (94.2) | 4043 (35.5) | 1494 (53.9) | 4779 (41.0) | **<0.001** |
| Witnessed OHCA | | 42268 (67.9) | 7755 (64.4) | 2657 (86.1) | 9741 (77.8) | **<0.001** |
| No-flow time (min) | | 11 [5; 18] | 8 [2; 15] | 11 [2; 21] | 10 [0; 18] | **<0.001** |
| Bystander CPR | | 27320 (43.9) | 6810 (56.5) | 2792 (90.5) | 10120 (80.9) | **<0.001** |
| Bystander CPR type | |  |  |  |  | **<0.001** |
|  | Ventilation only | 136 (0.5) | 10 (0.2) | 11 (0.4) | 37 (0.4) |  |
|  | Chest compression + ventilation | 3688 (14.0) | 976 (15.1) | 1774 (64.4) | 4516 (45.3) |  |
|  | Chest compression only | 22428 (85.4) | 5497 (84.8) | 969 (35.2) | 5425 (54.4) |  |
| Bystander AED application | | 2374 (5.2) | 1353 (14.2) | 892 (37.3) | 2848 (31.5) | **<0.001** |
| Bystander AED shock when applied | | 694 (31.4) | 681 (54.5) | 339 (41.6) | 691 (27.3) | **<0.001** |
| First MICU-recorded cardiac rhythm | |  |  |  |  | **<0.001** |
|  | Asystole | 51612 (83.5) | 8592 (72.1) | 2141 (69.9) | 9736 (78.7) |  |
|  | PEA | 3837 (6.2) | 894 (7.5) | 315 (10.3) | 1069 (8.6) |  |
|  | VF/pulseless VT | 4845 (7.8) | 1671 (14.0) | 335 (10.9) | 857 (6.9) |  |
|  | ROSC | 1486 (2.4) | 755 (6.3) | 271 (8.9) | 709 (5.7) |  |
| MICU-treated | | 44909 (72.2) | 9820 (81.5) | 2454 (79.5) | 7748 (61.9) | **<0.001** |
| Tracheal intubation | | 41248 (66.3) | 9305 (77.2) | 2256 (73.1) | 6985 (55.8) | **<0.001** |
| Epinephrine administration | | 40820 (65.7) | 8752 (72.8) | 2118 (68.9) | 6645 (53.3) | **<0.001** |
| Low-flow time (min) | | 30 [17; 42] | 30 [17; 44] | 30 [17; 45] | 25 [14; 40] | **<0.001** |
| ROSC | | 11785 (18.9) | 3468 (28.8) | 976 (31.6) | 2776 (22.2) | **<0.001** |
| Alive on arrival at hospital | | 9393 (15.1) | 3326 (27.6) | 900 (29.2) | 2241 (17.9) | **<0.001** |
| Alive at 30-day | | 2013 (3.2) | 1244 (10.3) | 367 (11.9) | 724 (5.8) | **<0.001** |
| CPC 1-2 at 30-day | | 1500 (2.4) | 951 (8.0) | 311 (10.2) | 575 (4.6) | **<0.001** |
|  |  |  |  |  |  |  |
| * Year 2011 is from July to December and year 2023 is from January to April. | | | |  |  |  |
| AED: automated external defibrillator; BLS: basic life support; CPR: bystander cardiopulmonary resuscitation; CPC: Cerebral Performance Category; MICU: mobile intensive care unit; OHCA: out-of-hospital cardiac arrest; PEA: pulseless electrical activity; ROSC: return of spontaneous circulation;  VF/pulseless VT: ventricular fibrillation/pulseless ventricular tachycardia. | | | | | | |
